# Supposed Polypus Nasi

**Published:** 1842-05

**Authors:** 


*Supposed Polypus Nasi.*—A young woman entered the Hospital of Clermont, under M. Fleury, to be operated on for polypus of the nasal fossæ, of which affection she had all the symptoms. At about 12 millimetres within each nostril, appeared a round whitish tumor which blocked up the passage. A stylet passed along the septum narium, penetrated easily into the nostril, but upon the external side of the nostril it was arrested by the pedicle of the tumor. An attempt was made to remove it in the ordinary way by the polypus forceps, but without success, and at each trial the patient complained of pain extending all over the head. Examining the tumor anew, the surgeon found that the excrescence taken for a polypus was only a prolongation of the pituitary membrane, which was folded over and thus reached the septum. It was the fold of membrane which in the normal state passes from the inferior turbinated bone, which from some unknown cause had become hypertrophied. The part was removed by a single cut of the curved scissors.

A similar case occurred at Hotel Dieu, under Dupuytren, in 1833. A M. Huckster presented herself with a red fungous tumor, projecting from the left nostril, and having the appearance of a polypus. Finding the base very large, some doubt was entertained as to the nature of this tumor. By the use of topical astringents the size was so much diminished that it was easy to see that the tumor was only a swelling of the mucous membrane lining the septum narium. Dupuytren insisted very strongly at his Clinique on the error of diagnosis to which such a case might give rise.—*N. Y. Med. Gaz.*

